# Designing and Evaluating an Autoverification RCV-Based System for Thyroid Function Profiles

**DOI:** 10.3390/diagnostics16030407

**Published:** 2026-01-27

**Authors:** Ran Gao, Chaochao Ma, Yingying Hu, Liangyu Xia, Fang Zhao, Qi Zhang, Liang Sun, Dawei Ai, Xinqi Cheng, Ling Qiu

**Affiliations:** 1 Department of Laboratory Medicine, Peking Union Medical College Hospital, Chinese Academic Medical Science and Peking Union Medical College, Beijing 100730, China; gaoran92@pumch.cn (R.G.); machaochao@pumch.cn (C.M.); huyingying@pumch.cn (Y.H.); xialy@pumch.cn (L.X.); zhaofang0305@126.com (F.Z.); zhangqipumch@163.com (Q.Z.); 2 Siemens Healthineers Diagnostics (Shanghai) Co., Ltd., 29F, 399 West Hai Yang Rd, Shanghai 200216, China; sun.liang@siemens-healthineers.com (L.S.); dawei.ai@siemens-healthineers.com (D.A.); 3 State Key Laboratory of Complex Severe and Rare Diseases, Peking Union Medical College Hospital, Chinese Academy of Medical Science and Peking Union Medical College, Beijing 100730, China

**Keywords:** autoverification, thyroid hormones, reference change value, turnaround time (TAT)

## Abstract

**Background/Objectives**: Autoverification systems integrated with fully automated analyzers and expert middleware can reduce manual review workload and improve turnaround time (TAT). This study aimed to develop an autoverification system for thyroid function profiles and to evaluate its performance in clinical practice. **Methods**: A total of 1,219,141 routine thyroid function test results collected from 1 January 2016 to 31 December 2020 were used to design the autoverification system. The system incorporated quality control checks, instrument error flags, limit range rules, delta check rules, and logical rules. Validation was performed using an independent dataset comprising 81,713 test results. **Results**: Twelve instrument error flags, a two-step delta check algorithm, one set of limit range rules, and ten logical rules were established. The overall autoverification pass rate was 75.2%. After system optimization, the overall pass rate increased to 77.8%, and the median laboratory TAT decreased from 122.1 min to 88.6 min. **Conclusions**: The autoverification system for thyroid function profiles significantly improved laboratory efficiency and reduced TAT. While RCV-based delta checks require prior results and are therefore not applicable to new patients, other autoverification rules remain active, and only results that cannot be autoverified are routed to manual review, ensuring safe and complete result verification. This system provides a practical framework for implementing autoverification in routine clinical chemistry testing.

## 1. Introduction

With the rapid advancement of laboratory automation and artificial intelligence, autoverification systems have emerged as an effective strategy for improving verification efficiency and reducing laboratory turnaround time (TAT) [1,2]. Such systems not only streamline workflow and decrease manual review burden but also enhance the detection of abnormal or inconsistent results, enabling laboratory professionals to focus their expertise on complex and clinically significant cases.

Thyroid hormones play essential roles in growth, development, and metabolic regulation. In China, thyroid disorders are highly prevalent: a national epidemiological survey reported that 50% of adults over 18 years have thyroid abnormalities, with 15.17% exhibiting thyroid dysfunction—including clinical and subclinical hyperthyroidism or hypothyroidism [3]. In addition, the prevalence of thyroid nodules in the healthy population has reached 36.9% (95% CI: 35.7–38.1%) [4]. The increasing incidence of thyroid diseases, together with rising health awareness and expanding access to laboratory testing, has substantially increased the test volume and workload in clinical laboratories.

Despite the clinical importance of thyroid function testing, relatively limited attention has been paid to comprehensive autoverification systems specifically designed for thyroid hormone assays in routine clinical workflows [5]. Existing reports vary widely in how delta check rules and time intervals are defined, and the logical relationships among thyroid hormones—although physiologically established—are complex and difficult to translate directly into automated decision rules. In addition, thyroid hormone interpretation is profoundly influenced by known physiological and clinical confounders, such as pregnancy, acute illness, and drug effects (e.g., biotin and glucocorticoids), which are often challenging to incorporate into automated systems. This variability and complexity underscore the need for standardized, data-driven, and clinically validated autoverification approaches.

In this study, we developed a real-world, data-driven autoverification system for thyroid function profiles, guided by the CLSI AUTO-10A and AUTO-15A international recommendations [6,7]. The system integrates analytical and clinical considerations, including population-specific data distribution, reference change values (RCVs), patient visit intervals, pharmacokinetics of commonly used medications, and expert input from endocrinologists. Our goal was to construct a robust, laboratory-tailored autoverification framework capable of improving efficiency while ensuring analytical and clinical reliability in thyroid function testing.

## 2. Materials and Methods

### 2.1. Subjects

This study was conducted in the clinical laboratory of Peking Union Medical College Hospital. A total of 1,219,141 thyroid hormone test results from both outpatients and inpatients between January 2016 and December 2020 were analyzed to establish autoverification rules. The dataset comprised 255,199 TSH, 226,776 T4, 226,771 T3, 255,197 FT4, and 255,198 FT3 measurements.

To validate the autoverification system, 503 electronically simulated cases and 81,213 real thyroid hormone test results collected between 7 March and 11 July 2022 were included.

The study was approved by the Ethics Committee of Peking Union Medical College Hospital, Chinese Academy of Medical Science (Approval No. I-25PJ1233, approved on 29 May 2025). Informed consent was waived for this study due to its retrospective nature.

### 2.2. Methods

#### 2.2.1. Instruments and Middleware

All thyroid hormones were measured using chemiluminescence immunoassay on Centaur XP immunoassay analyzers (Siemens Healthcare Diagnostics Inc., Tarrytown, NY, USA). The middleware systems, including Centralink (version 16) and Data Management Software (DMS; version R21.1), were also provided by Siemens Healthcare Diagnostics Inc. The Laboratory Information System (LIS) was supplied by Mediinfo (Hangzhou, China).

#### 2.2.2. Autoverification System Construction

Quality control check

Our laboratory routinely applies Levey–Jennings charts and Westgard multirule quality control procedures to monitor daily internal quality control (IQC). If IQC performance becomes unstable and cannot be corrected, a QC alarm is triggered. In such cases, patient results are automatically intercepted and routed to manual review.

Instrument error flags

Analyzer-generated alerts related to mechanical failures, reagent issues, and sample problems (e.g., incorrect specimen type, clotting, bubbles, insufficient volume, or non-numeric test results) are transmitted to the LIS. These flagged results are automatically withheld and directed to manual review.

Limit range check

We analyzed the distribution of thyroid function test results from all patients tested between 1 January 2015 and 31 December 2020. The 90th, 95th, and 99th percentile intervals of TSH, T3, T4, FT3, and FT4 concentrations were calculated to characterize population-based result distributions. These ranges were subsequently reviewed with clinical endocrinologists to establish analyte-specific limit ranges for autoverification.

Delta check

Delta is defined as the difference between current test results and prior test results [8]. If the delta exceeded the predefined limit, the result was withheld for manual review. In this study, the reference change value (RCV) for each analyte was used as the delta check criterion.

We retrospectively analyzed the interval between two consecutive thyroid function tests from patients with identical IDs between 1 January 2019 and 1 January 2020. The median or mean interval was used as the time window for delta check evaluation.

The formula to calculate the RCV is as follows:

$$\mathrm{RCV}\;=\;\sqrt{2}\times \;Z\;\times \;\sqrt{〔{\left({CV}_{A}\right)}^{2}+{\left({CV}_{I}\right)}^{2}〕}$$

where CVA is analytical coefficient of variation, CVI is within-subject biological variation, and Z is the coverage factor.

From 1 January 2016 to 1 March 2020, the analytical coefficients of variation (CVA) for the IQC of TSH, FT4, FT3, T4, and T3 were 7.1%, 7.0%, 5.0%, 6.5%, and 7.3%, respectively. The corresponding CVI for these analytes were obtained from the European Federation of Clinical Chemistry and Laboratory Medicine website and they were 21.2%, 7.7%, 6.0%, 6.4%, and 9.4%, respectively. After consultation with the endocrinologist, the RCV-based 95% significance threshold (Z = 1.96) was adopted as one of the delta check criteria.

Logical rules

We summarized thyroid function test result patterns from all patients and ranked their frequency. Together with endocrinologists, we identified patterns that should prompt additional attention, repeat testing, or evaluation for potential analytical errors or interferences. Test reports matching these patterns were intercepted and routed to manual review.

#### 2.2.3. Autoverification Validation Strategy

Electronically simulated case validation

Simulated cases were created for each rule category to verify the logic, sequence, and final decision of the autoverification system against manual interpretation. A total of 503 electronically simulated cases were constructed, including 196 cases of normal thyroid function, 132 cases of hyperthyroidism, 117 cases of hypothyroidism, 43 cases following thyroidectomy, 10 cases of hypopituitarism, 1 case of subclinical hyperthyroidism, 1 case of Mayer–Rokitansky–Küster–Hauser syndrome, as well as 3 cases representing analytical interference patterns, including two cases of heterophile antibody interference and one case of T4 autoantibody interference.

Trial operation phase verification

The system was deployed under real workflow conditions for five days. All results processed by the autoverification system were independently reviewed by senior technologists with more than 10 years of experience. The false-positive rate (system-intercepted but manually passed) and false-negative rate (system-passed but manually intercepted) were calculated.

Formal operation verification

The autoverification system was integrated into routine practice for 3 months and evaluated using at least 50,000 patient results. The automatic pass rate and turnaround time (TAT) reduction rate were calculated.

System optimization

Issues identified during prior validation phases—particularly from the non-passed results—were analyzed to refine the system. The optimized autoverification system was then re-evaluated during a three-week implementation period to assess automatic verification rate and TAT improvements.

### 2.3. Statistical Analysis

Data management and analysis were performed using R statistical software (version 4.0) and Microsoft Excel 2019. Normally distributed data were summarized as mean ± 2SD, whereas non-normally distributed data were presented as medians with interquartile ranges (P25–P75).

## 3. Results

### 3.1. Autoverification Rules

• Instrument error flags

Twelve error flags for the thyroid function profiles were set in the autoverification system. Detailed alarm information is presented in Table 1.

• Limit range check rules

The mean age of patients was 42.2 ± 15.4 years, and 68.4% of the patients were female. The 90th, 95th, and 99th percentile intervals for TSH, T3, T4, FT3, and FT4 are summarized in Table 2. After discussion with endocrinologists, the 99th percentile interval of each analyte was selected as the limit range for autoverification: TSH, 0.151–8.456 μIU/mL; FT3, 2.14–4.28 pg/mL; FT4, 0.80–1.81ng/dL; T3, 0.70–1.65 ng/mL; T4, 4.38–12.94 μg/dL.

• Delta check rules.

In the retrospective analysis of thyroid function testing data, the median interval between two consecutive examinations was 57.8 days. Levothyroxine sodium, a commonly prescribed medication for patients with thyroid disorders in endocrinology clinics, has an average half-life of approximately 7 days and is essentially eliminated from the body after 5–6 half-lives (35–42 days). Considering both the pharmacokinetic profile of levothyroxine and the observed patient revisit interval, the delta check time window was set to ≤60 days.

After consultation with the endocrinologist, the RCV-based 95% significance threshold (Z = 1.96) for each thyroid function test was adopted as one of the delta check criteria: which were set as follows:

Rule I (Percentage Delta Criterion)

The result passed the delta check if

Δ% = (∣ current – previous ∣)/previous × 100 < RCV

Rule II (Absolute Difference Criterion for Low Results)

If

Δ% > RCV and current ≤ Lower Reference Limit (LRL),

then the absolute difference criterion was applied. The result passed the delta check if

∣ current − previous ∣ < RCV × LRL

The detailed delta check criteria are summarized in Table 3.

• Logical rules

Based on the analysis of result patterns and expert consultation with endocrinologists, ten logical rules were established (Table 4). All ten rules were implemented simultaneously in the middleware and operated for one week. During this period, a total of 5668 thyroid function test specimens were processed.

Among them, 75 results (1.3%) were flagged by the logical rules, including those from 8 infants and 6 children. According to endocrinologist review, the flagged results did not align with the expected physiology of the hypothalamic–pituitary–thyroid (HPT) axis or showed patterns requiring clinical attention. These results were appropriately intercepted by the system and directed to manual review prior to report release.

### 3.2. Validation Results

In the electronic simulation phase (*n* = 503), the autoverification pass rate was 39.0% (196/503). All 307 intercepted cases displayed complete alert messages and system flags, fully consistent with the expected behavior.

A total of 5913 specimens were included in the trial operation validation. The overall autoverification pass rate was 76.8%. Among the 4540 results that passed autoverification, random sampling at a 1:30 ratio (151 cases) was subjected to manual review by two senior technologists, and no false-negative cases were identified. For the 1373 specimens intercepted by the system, random sampling at a 1:10 ratio confirmed the absence of false-positive cases.

During the official implementation phase (12 March to 12 June 2022), 57,488 specimens underwent autoverification. Of these, 43,224 passed the system checks, yielding a pass rate of 75.2%. Pass rates by analyte were 83.2% for TSH, 89.6% for FT3, 91.4% for FT4, 90.9% for T3, and 90.5% for T4. Among the specimens failing autoverification, random sampling at a 1:30 ratio identified 381 cases for manual review. Thirty-six cases (9.4%) were flagged solely because one or more thyroid function markers exceeded the predefined limit range rules, most commonly T3, followed by FT3 and T4. The remaining cases required retesting or additional review based on other laboratory indicators and clinical information.

**Figure 1:**
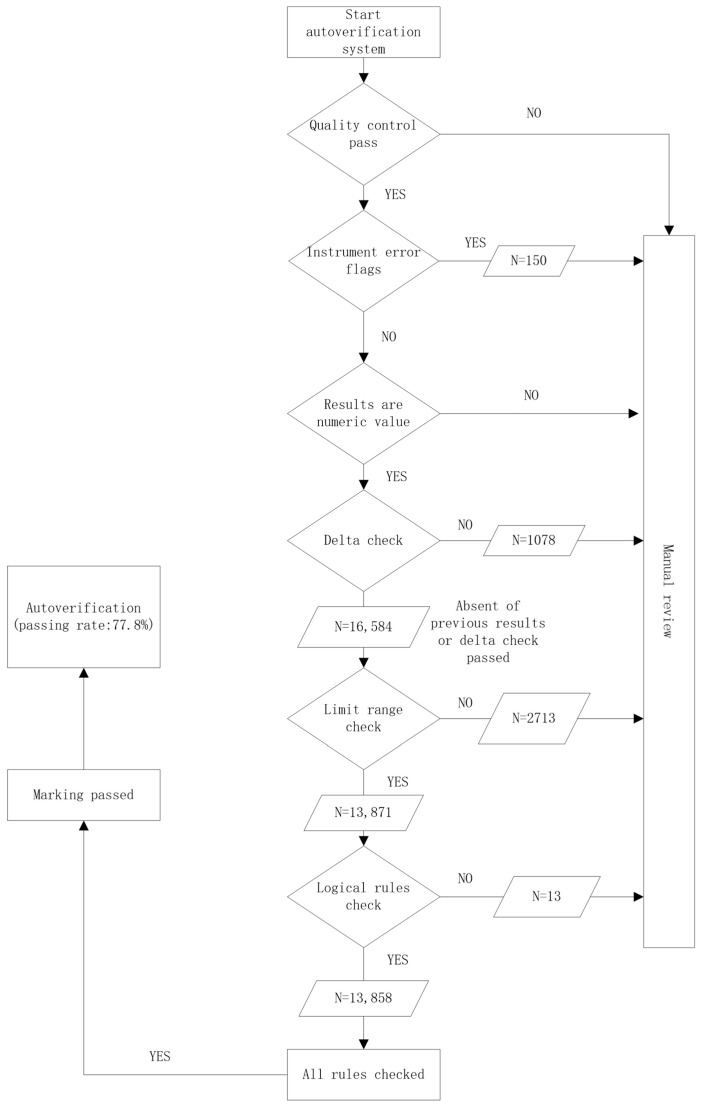
Autoverification decision tree based on sequential rule application. Quality control (QC) is applied as the initial gate. Only numeric results passing QC proceed to delta check, followed by range validation and logical rules. Results failing any step are routed to manual review, whereas only results passing all rule layers are automatically verified and released.

Following optimization of the autoverification system, 17,812 specimens were evaluated. The overall pass rate increased to 77.8%. In addition, the median laboratory turnaround time (TAT) decreased from 122.1 min to 88.6 min. Among cases that failed autoverification, the predominant rejection reason was violation of predefined result ranges, accounting for 70.0% of rejected cases. Historical data checks based on delta rules contributed to 26.0% of rejections. Instrument-related or quality control alerts accounted for 3.6%, while logical rule alarms represented only 0.4% of rejection events. The complete autoverification workflow is shown in Figure 1.

## 4. Discussion

Using 1,391,701 real-world thyroid function test results collected over six years, this study developed and validated a comprehensive autoverification system for thyroid function assays. The initial autoverification pass rate was 75.2%, which increased to 77.8% after system optimization. In parallel, the median turnaround time (TAT) decreased from 122.1 min to 88.6 min, demonstrating the system’s substantial impact on laboratory workflow efficiency.

We also observed that the limit ranges used in the autoverification rules differed from the analytical reference intervals. This discrepancy is expected because patient populations differ markedly among laboratories. Specialized facilities—such as pediatric hospitals or endocrine centers—often exhibit distinct distributions of thyroid hormone levels. Thus, autoverification limit ranges should be tailored to each laboratory’s patient demographics, particularly for age- and sex-dependent analytes. For example, FT3 concentrations in neonates and children are physiologically higher than in adults. As 96.7% of specimens in this study were from adults, separate limit range rules for special populations were not implemented.

Evaluation of a patient’s previous results is also critical in both manual and automated review. Delta check, defined as the comparison between current and previous results, may be expressed as delta difference, delta percent change, rate difference, or rate percent change [9]. In this study, we used delta percent change. However, no consensus exists regarding optimal delta thresholds or time intervals, and published methods vary widely [2,5,10,11]. For instance, Li et al. proposed thresholds of ±50% over 7 days for TSH, TT3, TT4, and FT3 and ±70% over 7 days for FT4 [5]. In this study, a uniform 60-day historical interval was applied across analytes. Although thyroid-related analytes such as TSH and FT4 differ in their biological response times, the selection of this interval reflects a balance between physiological considerations, including drug half-lives, and real-world laboratory practice, particularly patient revisit patterns and system applicability. Analysis of patient visit data over a two-year period demonstrated that a median of 57.8 days, with only 7.3% of patients underwent repeat testing within 14 days, 41.2% within 42 days, whereas 62.0% had repeat testing within 60 days. From a system-level perspective, the 60-day interval substantially increased the availability of prior results for delta checking, thereby enhancing the practical utility of autoverification. In contrast, shorter, analyte-specific intervals, while physiologically plausible, would markedly reduce system coverage and limit the contribution of historical comparisons in routine clinical workflows. Future studies may explore analyte-specific intervals in clinical settings with higher revisit frequencies or targeted patient subgroups.

We adopted the reference change value (RCV) as an objective, individualized threshold. RCV was calculated using the analytical variation observed in our laboratory in combination with established biological variation. Following consultation with endocrinologists, the 95% RCV for each analyte was selected as the decision limit. Because percentage differences can be exaggerated at low concentrations, we supplemented the delta percent rule with an absolute difference criterion. RCV is widely regarded as the minimum significant difference between serial measurements in an individual and therefore represents a suitable strategy for delta checking [12,13,14]. For broader implementation, laboratories may adopt published EFLM biological variation data when internal CVI estimates are unavailable, in combination with their own analytical imprecision, to derive locally applicable RCVs.

Thyroid hormones (FT4 and FT3) exhibit negative feedback on pituitary TSH secretion, and an inverse log-linear relationship between TSH and FT3/FT4 has been well documented [15,16,17]. However, in routine clinical practice, the relationship between TSH and thyroid hormone concentrations may be more complex than predicted by classical physiology. Notably, previous autoverification studies often applied simplified logical rules derived solely from hypothalamic–pituitary–thyroid (HPT) axis physiology rather than from empirical patient data [5,18,19,20]. In this study, we analyzed the distribution of FT3, FT4, T3, T4, and TSH result–pattern combinations across healthy individuals, outpatients, and inpatients. Incorporating these data-driven patterns, published evidence, and clinical consultation, we developed ten logical rules designed to detect physiologically implausible or clinically inconsistent combinations of thyroid function results. Although such atypical patterns occurred infrequently, they represent clinically important situations in which analytical interference, assay errors, or rare endocrine disorders may be present. During verification, endocrinologists reviewed all intercepted cases and confirmed that none conformed to normal HPT-axis regulation, supporting the validity of the logical rules.

The overall verification process included several stages. First, we assessed whether the autoverification system correctly identified abnormal results according to predefined rules. Second, we evaluated system performance and made targeted adjustments based on manual review findings. Third, we examined system stability in routine operation. During this stage, specimens failing autoverification were manually inspected, revealing that the limit ranges for T3, FT3, and T4 were overly restrictive and caused unnecessary result interception. After adjusting these limits to align with biological reference intervals, the final autoverification pass rate increased to 77.8% and the median TAT decreased to 88.6 min.

This study provides a practical framework for laboratories aiming to establish or improve autoverification systems. The approach used to construct delta check rules can be extended to other analytes. Because patient populations vary across institutions, each laboratory should determine its own limit ranges based on its local data. Development of logical rules requires careful consideration of physiological regulation and direct collaboration with clinicians.

Although automated clinical interpretation of thyroid function test results represents an important future direction, it is conceptually distinct from laboratory autoverification. Autoverification focuses on analytical validity, result plausibility, and workflow safety, whereas clinical interpretation requires comprehensive integration of individual patient context. Despite recent advances in artificial intelligence, the automation of clinical commenting for thyroid function tests remains challenging due to the complex influence of multiple physiological and clinical factors, particularly medication-related variables. In this context, robust autoverification serves as a necessary foundational step prior to any higher-level automation of clinical interpretation.

Several limitations should be acknowledged. This study aimed to evaluate the overall performance, safety, and real-world applicability of a unified autoverification rule set, rather than to develop population- or condition-specific strategies; therefore, stratification by age, sex, pregnancy status, or medication use was not incorporated at this stage. Additional analyses showed that the mean age and female proportion among cases that failed autoverification were comparable to those of the overall validation cohort, suggesting no systematic age- or sex-related bias. Given the known physiological effects of pregnancy on thyroid function, particularly FT4, a conservative unified approach was intentionally adopted so that atypical or borderline results are more likely to be routed to manual review, thereby enhancing result safety. Medication use was not included due to the lack of structured data in the current laboratory information system and remains a focus for future development.

## 5. Conclusions

We developed and validated an autoverification system for thyroid function profiles using large-scale real-world data, which significantly improved laboratory efficiency and reduced turnaround time. By adopting reference change values (RCVs) as historical data check rules, the system accounts for both the biological variability of thyroid function analytes and the analytical performance of the laboratory, thereby contributing substantially to safe and effective autoverification. By reducing manual workload and audit-related errors, this system enables laboratory professionals to focus more effectively on complex or clinically significant cases and provides a practical framework for implementing autoverification in routine clinical chemistry practice.

## Figures and Tables

**Table 1:** Rules of Instrument error flags.

SN	Code	Rules’ Explanation	Resolution
1	IS-1	The result was below or above the measuring interval	Manual review
2	IS-2	The result was below or above the linearity	Manual review
3	IS-3	The result was above the dilution setpoint in the test definition	Manual review
4	IS-4	The kit failed to be discarded or the kit did not arrive at the specified location	Error Flag
5	IS-5	Reagent chamber temperature or humidity exceeds the set range	Manual review
6	IS-7	Reagent batch number expired	Error Flag
7	IS-8	The specimen has clots or insufficient specimen size	Manual review
8	IS-9	Specimen bar code error	Error Flag
9	IS-11	Sample pin fault	Error Flag
10	IS-12	Loading of sample adding suction head failed	Error Flag
11	IS-13	Hydraulic insufficiency	Error Flag
12	IS-14	Reagent needle and sample needle cleaning module is faulty	Error Flag

**Table 2:** The distributions of TSH, FT3, FT4, T3 and T4.

	TSH (μIU/mL)	FT3 (pg/mL)	FT4 (ng/dL)	T3 (ng/mL)	T4 (μg/dL)
99th percentile interval	0.151–8.456	2.14–4.28	0.80–1.81	0.70–1.65	4.38–12.94
95th percentile interval	0.257–6.874	2.3–4.07	0.88–1.72	0.76–1.54	5.00–12.20
90th percentile interval	0.42–5.57	2.42–3.86	0.93–1.62	0.80–1.44	5.40–11.40
RI ^1^	0.380–4.340	1.80–4.10	0.81–1.89	0.66–1.92	4.30–12.50

^1^ reference interval.

**Table 3:** The delta check rules of thyroid function profiles.

	TSH	FT4	FT3	T4	T3
CVA	7.1%	7.0%	5.0%	6.5%	7.3%
CVI	21.2%	7.7%	6.0%	6.4%	9.4%
95% RCV ^1^	62.0%	28.8%	21.7%	25.3%	33.0%
99% RCV	73.4%	34.1%	25.6%	31.3%	41.9%
Cut-off of absolute difference	0.31	0.26	0.49	2.35	0.26

^1^ reference change value.

**Table 4:** The logical rules for thyroid function profile autoverification.

	Logical Rules
1	When TSH decreases, T3, T4, FT3, FT4 cannot decrease in any item
2	When TSH increases, T3, T4, FT3, FT4 cannot increase in any item
3	When FT3 decreased, the results of T3, T4 and FT4 should be normal or decreased, without increasing
4	When FT3 increases, the results of T3, T4 and FT4 should be normal or increased, without decreasing
5	When FT4 decreased, the results of T3, T4 and FT3 should be normal or decreased, without increasing
6	When FT4 increases, the results of T3, T4 and FT3 should be normal or increased, without decreasing
7	When T3 decreases, the results of T4, FT4 and FT3 should be normal or decreased, but not elevated
8	When T3 is elevated, the results of T4, FT4 and FT3 should be normal or elevated, but not decrease
9	When T4 is reduced, the results of T3, FT4 and FT3 should be normal or decreased, but not elevated
10	When T4 is elevated, the results of T3, FT4 and FT3 should be normal or elevated, but not decreased

## Data Availability

The data presented in this study are available on request from the corresponding authors due to ethical and policy restrictions.

## Abbreviations

The following abbreviations are used in this manuscript:

TSH	thyroid-stimulating hormone
T3	triiodothyronine
T4	thyroxine
FT3	free triiodothyronine
FT4	free thyroxine
LIS	Laboratory information system
RCV	Reference change value
TAT	Turnaround time
